# Harvesting the Energy of Multi-Polarized Electromagnetic Waves

**DOI:** 10.1038/s41598-017-15298-5

**Published:** 2017-11-07

**Authors:** Thamer S. Almoneef, Faruk Erkmen, Omar M. Ramahi

**Affiliations:** 1 Department of Electrical Engineering, Prince Sattam bin Abdulaziz University, Al-Kharj, 11942 Saudi Arabia; 2 Department of Electrical and Computer Engineering, Waterloo, N2L3G1 Canada

## Abstract

We present the idea and design of a dual polarized metasurface for electromagnetic energy harvesting. A 4 × 4 super cell with alternating vias between adjacent cells was designed to allow for capturing the energy from various incident angles at an operating frequency of 2.4 GHz. The collected energy is then channeled to a feeding network that collects the AC power and feeds it to a rectification circuitry. The simulation results yielded a radiation to AC and an AC to DC conversion efficiencies of around 90% and 80%, respectively. As a proof of concept, an array consisting of 9 super cells was fabricated and measured. The experimental results show that the proposed energy harvester is capable of capturing up to 70% of the energy from a planewave having various polarizations and converting it to usable DC power.

## Introduction

Metamaterials can be made by assembling electrically-small resonators which can take different shapes and composite materials. The fact that a metamaterial surface can be engineered to produce an effective medium having simultaneously negative permeability and permittivity has ignited a number of unprecedented applications in various frequency bands from acoustics^1,2^ to the visible regime^3,4^. Such applications include cloaking^5,6^, energy harvesting^7–10^, negative index of refraction^11^, perfect lensing^12^, and perfect absorption^13^. In metamaterial perfect absorbers, the unit cells are designed in such a way to produce a surface impedance matched to free space. This can be achieved by tuning the $\mu$ and $\varepsilon$ of the medium such that the refractive index is $n=1$. In addition, a lossy dielectric substrate is used to dissipate the absorbed energy.

In a recent article, a metamaterial surface inspired by the perfect absorption concept was introduced for electromagnetic energy harvesting^7^. In the paper, an array of $13\times 13$ electrically-small Electric Inductive-Capacitive (ELC)^14^ unit cells was used to fully absorb an incoming plane wave at 3 GHz. Unlike metamaterial absorbers, here the absorbed energy is mainly dissipated across resistive loads placed at the feed of each resonator. However, the work was focused on maximizing the radiation to AC conversation efficiency. The array was also sensitive to the polarization of the incoming wave, maximizing the efficiency for only one single polarization. What is important for electromagnetic microwave energy harvesting/scavenging is to maximize the captured ambient energy from all polarizations. Moreover, instead of resistive loads, a rectification circuit has to be implemented to maximize the radiation to DC efficiency forming a full rectenna (rectifying antenna) system^15–19^.

In this paper, we propose a dual polarized electromagnetic energy harvester using an array of ELC resonators to form a metamaterial medium. The radiation to AC and the AC to DC conversion efficiencies of the array are studied numerically and experimentally.

## Design Methodology

**Figure 1:**
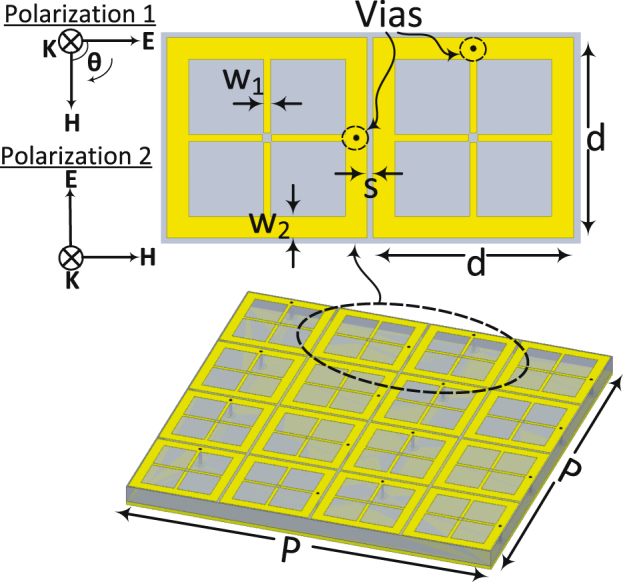
A schematic showing the proposed super cell of the metamaterial harvester. The inset shows two cells with different via positioning. The inset also shows the two proposed incident wave polarizations referred to in all sections of the paper.

A unit cell of the proposed dual polarized harvester is shown in Fig. 1. The unit cell consists of $4\times 4$ cells forming a super cell. The super cell is hosted on top of a 3.175 mm thick RT/duroid 5880 Rogers dielectric substrate having a loss tangent of $tan\delta$ = 0.0009 and a dielectric constant of ${\varepsilon }_{r}$ = 2.2. The super cell is backed by a ground plane to block the incident wave from transmitting through the cell. Each cell contains a load resistance placed between the ground plane and the top layer through a via. The position of the via is alternated for each adjacent cell to allow for capturing the incoming wave from various incident angles as shown in the inset of Fig. 1. Each cell has dimensions of d = 17 mm, ${w}_{1}$ = 0.6 mm, ${w}_{2}$ = 1.85 mm, s = 0.5 mm and copper thickness of t = 35 *μ*m. The side length of the super cell is P = 70 mm having a footprint area of A = 49 cm^2^.

The choice of the number of cells contained within the super cell is specifically important for the feeding network. One can choose a super cell that contains only two cells as showing in the inset of Fig. 1. However, by doing so the channeling circuitry (which will be discussed later in further details) will be different and more intricate. The main advantage of grouping cells within a super cell is to reduce the feeding ports. By designing a super cell that contains 16 cells where each 8 cells is responsible for capturing one single polarization, the 16 cells can be fed by only 2 feeding networks, simplifying the channeling mechanism of the collected AC power significantly.

### Numerical Analysis of The Radiation to AC Conversion Efficiency

**Figure 2:**
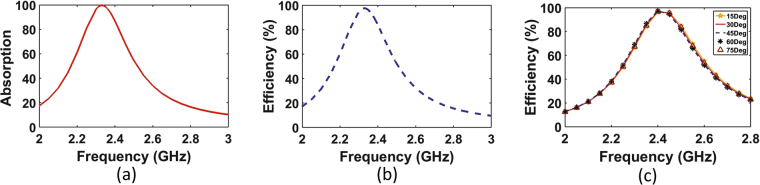
A simulation study of the super cell showing (**a**) the absorption and (**b**) the power across the resistive load both as a function of frequency. At the resonance frequency of around 2.4 GHz, 98% of the power is trapped within the resistive load. Both figures represent the results obtained for the two polarizations shown in (Fig. 1). However, since both curves perfectly overlap due to the high symmetry of the super cell, only one curve is shown in each sub figure. Sub figure (**c**) shows the power across the resistive loads of the super unit cell at various polarizations, where the angle $\theta$ is measured by rotating the E field vector clockwise as shown in the inset of Fig. 1.

The unit cell was studied using the full wave simulator HFSS^20^. The super cell was placed in a waveguide where the lateral walls were assigned periodic boundary conditions in order to simulate an infinite periodic surface. The waveguide was excited by a Floquet port having a power level of 1 W and two incident wave polarizations as shown in Fig. 1. The scattering parameters for each polarization were extracted to compute the power absorption of the super cell. The absorption of the super cell can be obtained by $A(\omega )$ = 1 − $| {S}_{11}{| }^{2}$ − $| {S}_{21}{| }^{2}$ where the transmission coefficient $| {S}_{21}{| }^{2}=0$ due to the presence of the ground plane. Figure 2 shows the absorption of the unit cell as a function of frequency.

When each cell is terminated by a load resistance of R = 175 ${\rm{\Omega }}$, the super cell experiences full absorption of the incoming wave at the resonance frequency of around 2.4 GHz. It is also critical to concentrate the loss within the load rather than the substrate material for energy harvesting applications. This trapped energy is later to appear across the leads of a diode or a rectification circuitry for AC to DC power conversion. To achieve such condition, the host material must have a very low loss (i.e. low $tan\delta$) at the operating frequency to minimize the dielectric losses. In addition, a load resistance must be used to mimic a rectification circuit and to allow for a dissipation mechanism for the absorbed energy within the cells. This load resistance is selected carefully such that the input impedance of the cell seen from the feeding port or the load location is matched to the impedance of the cell. This is similar to the input impedance of a probe-fed patch antenna where the input impedance of the antenna depends on the location of the via, except that here the cells are highly coupled where the input impedance can be modified by controlling the coupling effect in addition to the feeding location. The choice of this impedance is also critical for the rectification circuitry as it dictates the impedance of the channeling network and the matching network between the diode and the energy collector.

**Figure 3:**
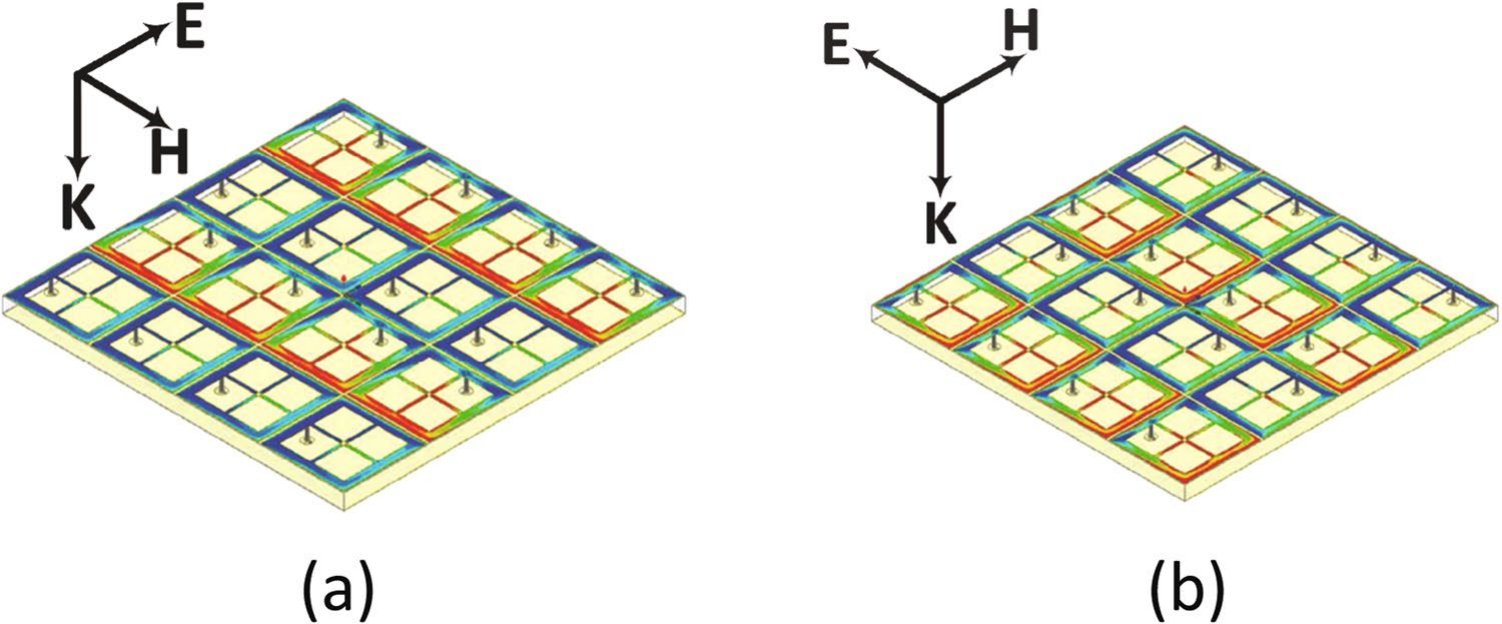
The E-field magnitude plot across the surface of the super cell for (**a**) polarization 1, and (**b**) polarization 2. From the plot, the field is pronounced across only 8 cells per polarization as illustrated by the red color.

Figure 2(b) shows the efficiency of the super cell as a function of frequency. For both the absorption (Fig. 2(a)) and the efficiency (Fig. 2(b)) of the super cell, the curves represent both polarizations as shown in Fig. 1. In addition to the two orthogonal polarizations used in the simulation, the super cell was tested for various incident angles ($\theta$) measured with a clockwise rotation of the E field vector as shown in Fig. 1. The angle ($\theta$) was varied from 15° to 75° in increments of 15° as illustrated in Fig. 2(c). From the results we can observe that all the efficiency vs. frequency curves for all the incident angles overlap showing that the harvester can receive the electromagnetic energy from the incoming plane wave equally regardless of the orientation of the parallel E field vector. This efficiency is calculated by taking the ratio of the total energy dissipated across all the 16 resistors of each cell to the incident power. It is interesting to note here that 98$\%$ of the incident power is dissipated across the load resistors. However, only 8 resistors are responsible for absorbing the energy for each polarization. This can be understood by the electric field plot across the surface of the super cell as shown in Fig. 3(a) for polarization 1 and Fig. 3(b) for polarization 2. It is interesting to note here that for each polarization only 8 cells contain vias at the arms that are parallel to the incident electric field direction. Thus, one expects the cells to absorb half of the total incident energy at each polarization. This is true if the cells were weekly coupled. However, since the cells are strongly coupled, the energy from the cells that contain vias in the arms that are orthogonal to the direction of the incident electric field is coupled to the other 8 cells which contain vias in the arms that are parallel to the electric field direction. This is true for both polarizations, thus the super cell experiences full absorption at each polarization. This is clear from the field plot where the 8 cells that contain vias in the arms that are parallel to the electric field experience high field concentration for both polarizations as indicated by the red color. This was observed in the simulation where for each polarization, 98% (please see Fig. 2(b)) of the dissipated power was due to the aggregate energy dissipation across only 8 resistors. This shows that the electric current flowing on the other 8 cells that did not contribute to the total dissipated energy was coupled to the cells that did contribute to the total absorbed energy. Such feature simplify the feeding network such that the energy is tapped from only 8 cells per polarization rather than from all the 16 cells. This significant simplification in the channeling mechanism results in reduction in the power losses in the feeding network since less copper traces are used.

To channel the energy to the desired load, each cell can be terminated by a diode instead of the resistor. Thus, each individual cell converts the incident electromagnetic energy directly to DC. Then the DC power contribution from each cell can be combined through DC buses to a single load as was done in a recent work^16^. In this method^16^, the number of diodes were equal to the number of cells in the system. This can be in the range of hundreds depending on the size of the harvester, thus the total cost of the system will increase. In addition, the AC to DC conversion losses increase linearly with the number of diodes. Other channeling mechanism reported recently use a feeding network to channel all the collected AC power to a single diode or rectification circuitry^21^. In these methods the AC losses of the feeding network, especially for large footprints, can be significant.

**Figure 4:**
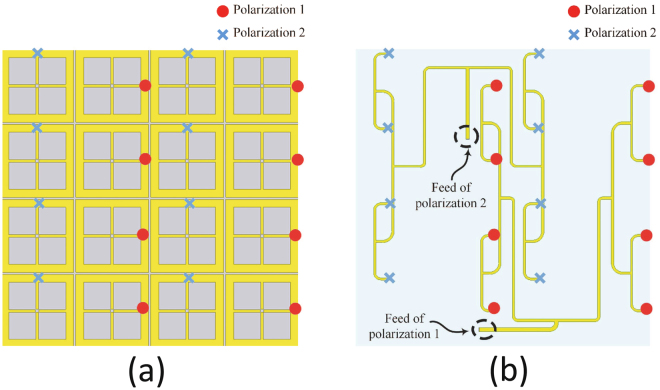
A schematic showing the proposed feeding network for the super cell. The feeding networks for the two orthogonal polarizations, 1 and 2, are not identical to allow for sufficient space for the rectifiers to be integrated on the same layer. (**a**) Back view. (**b**) Front view. The o and x marks show the location of the vias used for polarization 1 and 2.

In this paper, we propose a new channelling mechanism that combines both AC and DC channeling networks as shown in Fig. 4. The AC power collected by the 8 cells for each polarization is combined in three stages to one single feed. The two resulting feeds are to be connected to two separate rectifiers for AC to DC power conversion. The AC power combining circuit for each polarization is not necessarily the same due to the limited space available for each super cell.

The feeding network is placed on top of an RO4003 Rogers dielectric substrate having a thickness of 1.524 mm, loss tangent of $tan\delta$ = 0.0027, and a dielectric constant of ${\varepsilon }_{r}$ = 3.55. The feed network was attached at the bottom of the super cell material forming three separate layers with two different substrate materials. The ground plane is sandwiched between the two materials and the cells are connected to the feed network through via holes. The feed network along with the super cell were simulated as a one system. Each feed location (shown in Fig. 4(b)) was assigned a lumped source with an impedance of R = 175 Ω. The periodic boundary condition was applied to the lateral walls similar to the boundary condition assigned for the super cell except that the top and bottom sides of the waveguide were assigned absorbing boundary conditions instead of excitation ports to analyse the system in the transmitting mode. The transmitting mode analysis is performed to find the directional input impedance of the super cell looking from the feed location as showing in Fig. 4(b). This impedance is used later to match the rectifier with the super cell for each polarization.

**Figure 5:**
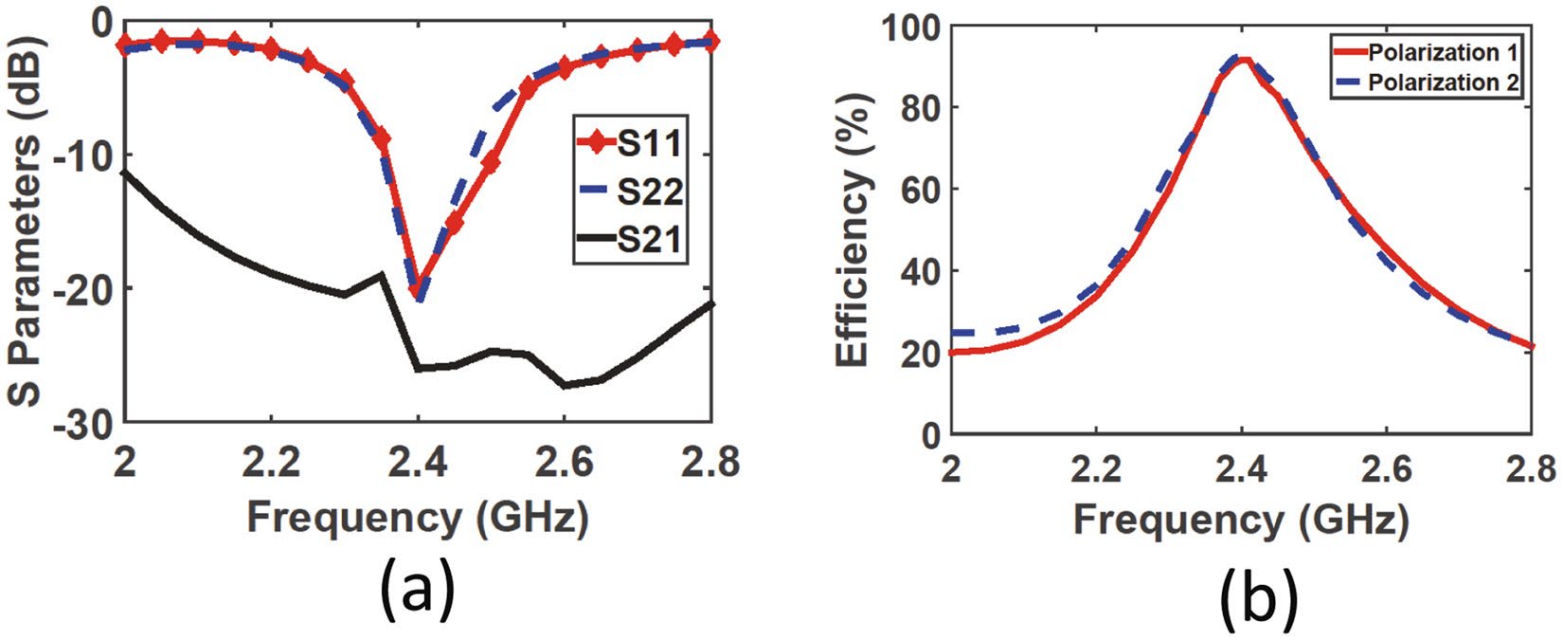
A simulation plot of (**a**) the scattering parameters showing the reflection coefficient of each polarization (${S}_{11}$ and ${S}_{22}$) and the transmitting coefficient (${S}_{21}$) between the two feeds (see Fig. 4) and (**b**) the radiation to AC efficiency of the super cell when connected to the feeding network through via holes for both polarizations.

The simulated scattering parameters are shown in Fig. 5(a), where ${S}_{11}$ and ${S}_{22}$ are the reflection coefficients of polarizations 1 and 2, respectively. Both reflection coefficients show good matching at the resonance frequency with a value of less than −20 dB for each polarization. The transmission coefficient ${S}_{21}$ between the two feeds is almost negligible indicating that the cross talk between the two feeds is minimal. This is critical so that the feeding transmission lines for each polarization does not effect the impedance of each feeding network, thus the rectifier will always experience the same input impedance from the super cell side.

The complete system is then analysed in the receiving mode to test the amount of energy captured from each incident polarization. The two feeds where terminated by R = 175 Ω and the super cell was excited by an incident wave having a normalized power of 1 W. The radiation to AC efficiency of both polarizations is shown in Fig. 5(b). For both polarizations, the radiation to AC efficiency of the unit cell was 92$\%$ at the resonance frequency of 2.4 GHz. This shows that the loss of the feeding network was 5$\%$ since the radiation to AC efficiency of the super cell without feeding is 97$\%$ as reported earlier.

## Numerical Analysis of The AC to DC Conversion Efficiency

**Figure 6:**
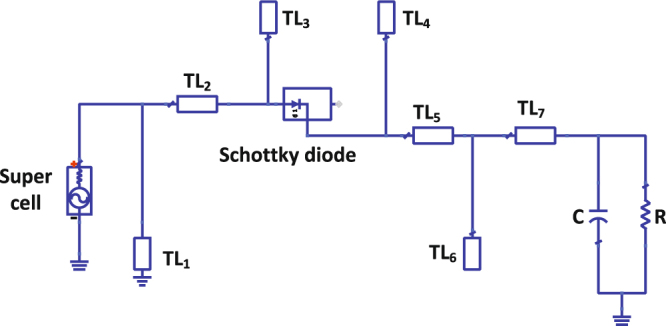
A circuit schematic showing the rectifier circuit designed in ADS. The layout is for both polarizations but with different parameters for each polarization as listed in Table 1. There is a total of 7 microstrip transmission line segments where TLn refers to the nth segment.

Taking into consideration the results obtained for the radiation to AC power conversion, a rectification circuitry was designed to build a full rectenna system. A rectifier circuit was simulated in ADS for each polarization. In the simulation, the Z parameters that was obtained from the full-wave numerical simulation for each polarization were extracted and imported into ADS. Then a 1-tone frequency power source was used to mimic the super cell looking from the feeding location (see Fig. 4) for each polarization. The Z parameters were inserted into a Data Access Component (DAC) and then it was assigned as the input impedance of the power source. This way the power source will have a different impedance at every frequency to mimic the real impedance of the super cell for each polarization. The rectifier circuit schematic built in ADS is shown in Fig. 6. The rectifier circuit designed for each polarization have the same circuit layout but with different transmission lines’ length and width. The length (L) and width (W) for each transmission line is given in Table 1 where the subscript number refers to the transmission line numbering in accordance with Fig. 6.

**Table 1:** The rectifier parameters of the circuit layout of Fig. 6 for both polarizations. All the listed parameter are in (mm).

Parameter	Polarization 1	Polarization 2
L1/W1	3.20/0.50	5.00/0.50
L2/W2	2.01/1.00	1.02/0.97
L3/W3	6.56/4.98	7.28/2.45
L4/W4	1.23/3.00	0.40/4.50
L5/W5	1.34/0.57	8.93/0.40
L6/W6	4.24/1.70	1.98/2.98
L7/W7	3.70/1.94	2.02/3.83

**Figure 7:**
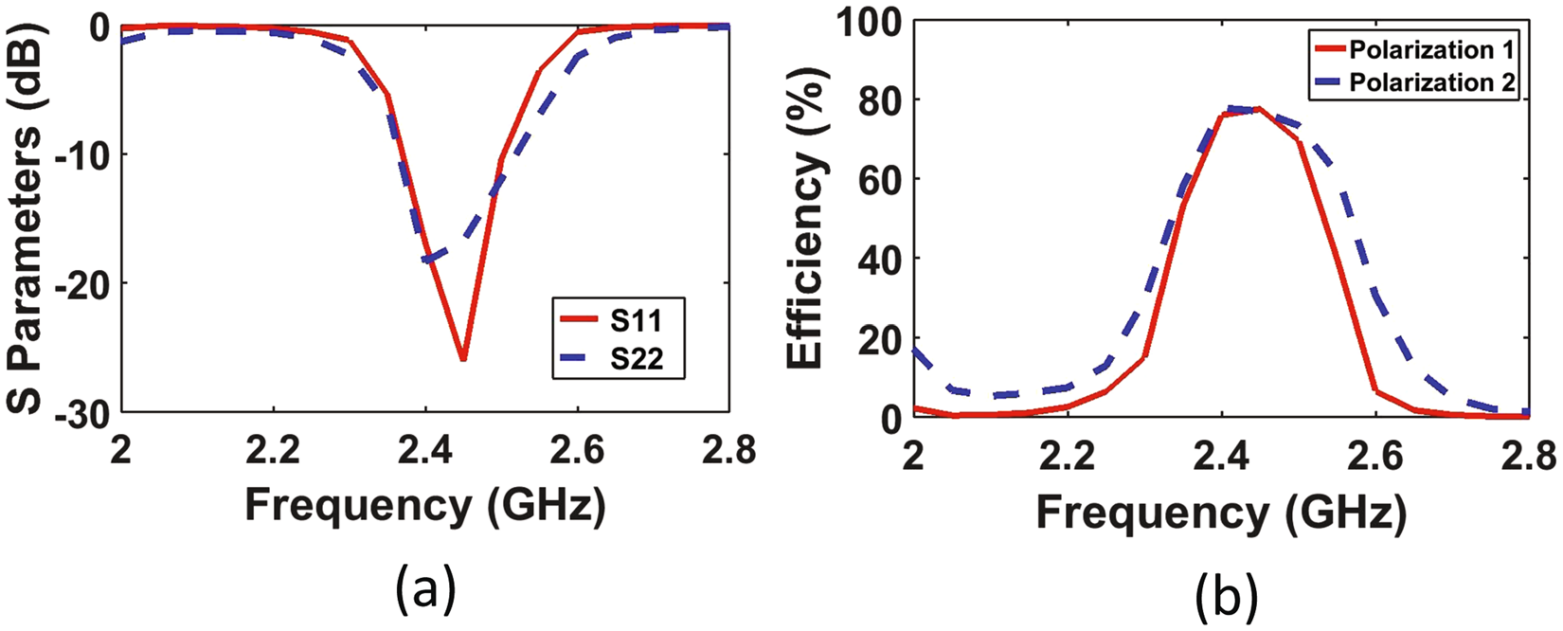
A simulation results showing (**a**) the reflection coefficient of the rectifier for each polarization (${S}_{11}$ and ${S}_{22}$) using the LSSP simulator in ADS and (**b**) the AC to DC conversion efficiency of the rectifier for each polarization as a function of frequency.

In the simulation, the Harmonic Balance (HB) simulator was used to analyse the rectifiers due to the presence of the nonlinear diodes. The circuit contains a series transmission line, shorted stub and open circuited stub between the super cell and the diode. A series HSMS 2860 Schottky diode was used for its low turn on voltage of 0.28 V and fast switching speed at the frequency of operation of 2.4 GHz. In addition, two open circuited stubs and two series transmission lines were used between the diode and the RC circuit to ensure a good match between the diode and the load resistance and to filter out all the higher order components of the waveform before reaching the load. A shunt capacitor of 120 pF was selected to appear as a short circuit for higher frequencies and an open circuit for the rectified DC power. The rectifier was designed to have an impedance conjugate to that of the super cell for each polarization in order to maximize the power transfer between the energy collector and the rectifier. The Large Signal S-Parameters (LSSP) simulator was used in ADS to analyse the reflection coefficient of the rectifier for each polarization as shown in Fig. 7(a). The diode’s input impedance and performance is significantly dependent on the input power level. For low power levels, small signal analysis can be used to obtain accurate results of the diode’s response. However, once higher power levels are used to drive the diode, LSSP analysis must be used for accurate prediction of the diode’s behaviour. The reflection coefficients of the rectifier for both polarizations show a well matched condition around the resonance frequency. The obtained profile for both rectifiers shown in Fig. 7(a) is similar to the one obtained by the full-wave simulation (see Fig. 5(a)) for the super cell. This is due to the fact that the 1-tone frequency power source in ADS was assigned a frequency dependant input impedance that mimics the input impedance of the super cell.

In addition, the AC to DC power conversion of the rectifier was analysed for each polarization as shown in Fig. 7(b). The efficiency was calculated by taking the ratio of the power dissipated across the load to the input power assigned at the 1-tone frequency power source. The simulated efficiency shows that the rectifiers for both polarizations were capable of converting around 80$\%$ of the AC power collected by the super cell to DC power across the load resistance of R = 1500 Ω. Furthermore, there is at least 10$\%$ of an inevitable intrinsic losses attributed to the power conversion process by the diode.

**Figure 8:**
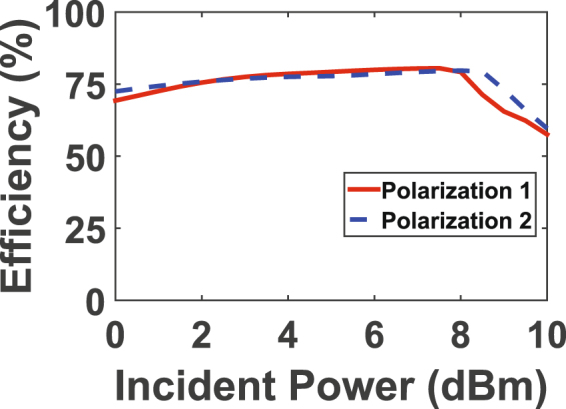
Simulation results showing the radiation to DC efficiency of the rectifier for each polarization as a function of input power level.

The effect of the input power level for both rectifiers on the AC to DC efficiency was also analysed to ensure that the rectifiers perform at their maximum efficiency. The rectifier was fed by a sweep of input power to analyse the magnitude of the AC to DC power conversion as a function of the input power. The simulated input power sweep as a function of AC to DC efficiency is shown in Fig. 8 for both polarizations. From both curves, the rectifiers experience the highest efficiency at around 9 dBm of input power.

## Experimental Verification

**Figure 9:**
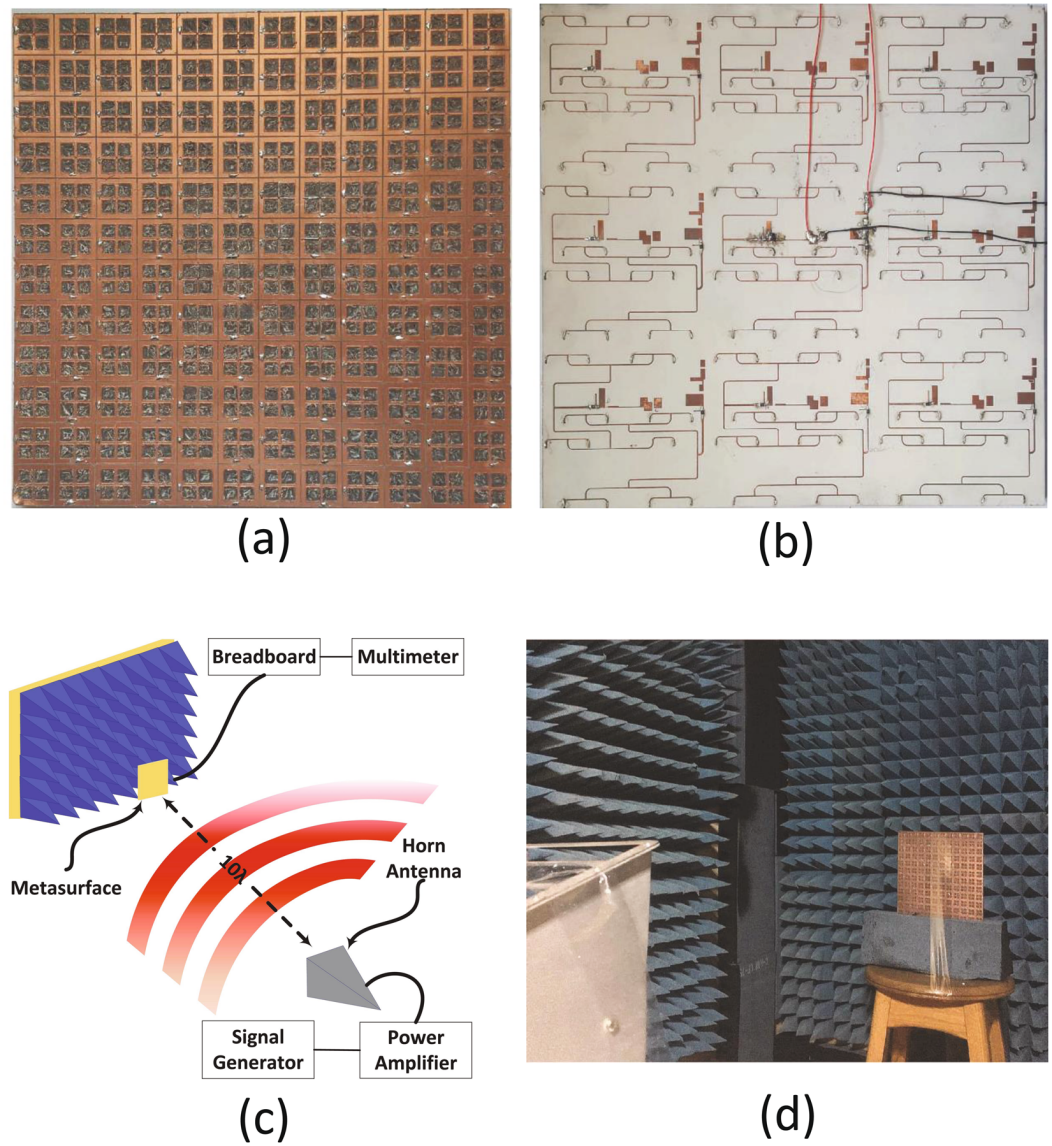
A photograph of the fabricated rectifying metasurface (**a**) top layer and (**b**) bottom layer showing the feeding network along with the rectifier for both polarizations. Sub-figure (**c**) shows a schematic of the experimental setup used in the experiment hosted in side an anechoic chamber. Sub-figure (**d**) shows the experimental setup inside an anechoic chamber.

In light of the results obtained from the full-wave simulation for the radiation to AC analysis and the results obtained from ADS for the AC to DC analysis, a full rectenna system was built as shown in the photos of Fig. 9(a) and (b). Nine super cells were built with a total of 144 cells on RT 5880 Rogers duroid substrate material. The feeding networks along with the rectifiers were built on an RO 4003 material. Both materials were stacked together to form a system with three layers and two substrates. The middle super cell was terminated by a Schottky diode, a shunt capacitor and a variable resistor for each polarization. The other 8 super cells were terminated by the matched load resistance of R = 175 ${\rm{\Omega }}$. The experiment was performed in an anechoic chamber with a setup as shown in Fig. 9(c). A photo of the measurement setup is shown in Fig. 9(d).

**Figure 10:**
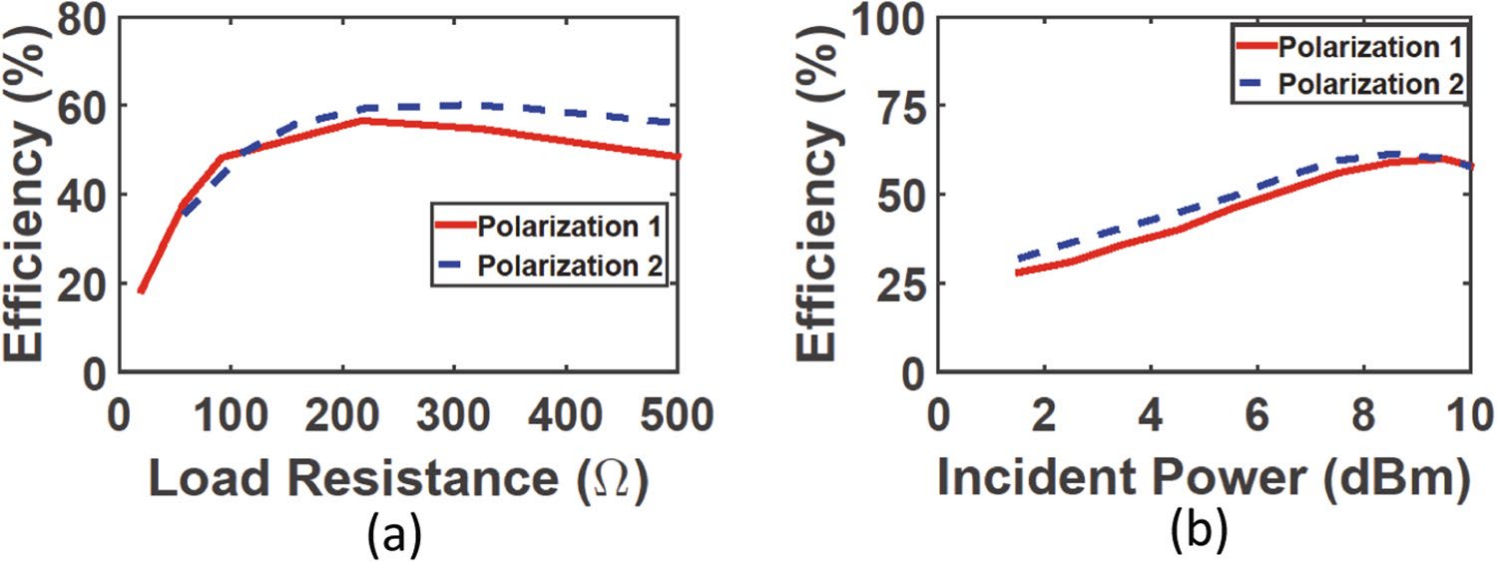
Measurement results showing (**a**) the efficiency of the rectifier for each polarization as a function of load resistance (**b**) the efficiency of the rectifier for each polarization as a function of input power level.

In the measurement, a 10 dBi gain horn antenna was used as a transmitting antenna. The horn antenna was fed with an E4438C keysight ESG Vector Signal Generator having a maximum power and frequency of 25 dBm and 6 GHz. To control the amount of power fed to the horn antenna, a Mini Circuits High Power Amplifier ZHL-16W-43-S is connected between the horn antenna and the signal generator. The metasurface was placed 1 m away from the transmitting horn antenna. The voltage across each variable resistor for each polarization was measured separately using a multi-meter. The radiation to DC power conversion efficiency of the super cell was calculated by the following equation^7,9^:

1 $$\eta =\frac{{P}_{out}}{{P}_{in}}$$

where ${P}_{in}$ is the total time-average power incident on the super cell. ${P}_{in}$ was calculated as:

2 $${P}_{in}={P}_{T}{G}_{T}(\frac{A}{4\pi {R}^{2}})$$

where ${P}_{T}$ and ${G}_{T}$ are the power and gain of the transmitting antenna, respectively, and $A$ represents the entire footprint or area of the super cell which is A = 49 cm^2^. In addition, ${P}_{out}$ is the total DC power developed across the resistive loads of each rectifier. It is critical to ensure that the rectifier is operating at its optimal incident power level and load resistance for maximum power delivery between the super cell and the rectifier. Therefore, the load resistances across the rectifier of polarization 1 and 2 were swept and the radiation to DC power conversion efficiency was measured at each input power level. Figure 10(a) shows that both polarizations experience a peak efficiency at approximately 250 Ω.

The incident power was then swept as a function of efficiency while both rectifiers are terminated by the optimal resistance of 250 ${\rm{\Omega }}$. The curves for both polarizations in Fig. 10(b) show that the efficiency is maximum when the incident power was around 9 dBm. Here, the incident power is referred to as the real power falling on the surface of the middle super cell which is calculated by the product of the footprint area (A) and the power density at the surface of the harvester.

**Figure 11:**
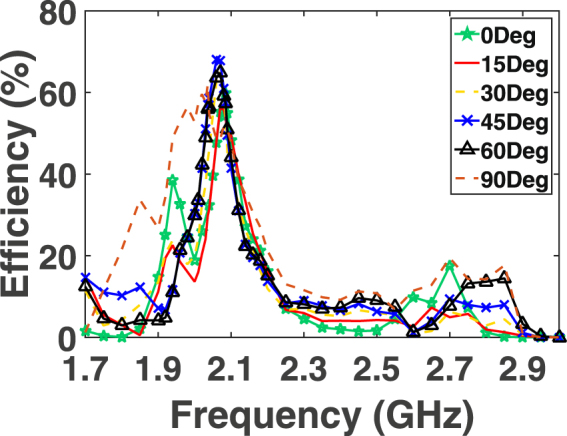
Measurement results showing the efficiency of the rectifier as a function of frequency for various incident angles.

The efficiency of the rectenna system is then analysed as a function of frequency. In this measurement step, the incident power and the load resistance were fixed at the optimal values obtained above. In addition to the two incident polarizations proposed in Fig. 1, four additional incident angles of 15°, 30°, 45° and 60° were also tested. The angle $\theta$ is measured with respect to the clockwise rotation of the E field vector of polarization 1 as shown in Fig. 1. Figure 11 shows the radiation to DC efficiency as a function of frequency for six incident angles. In the figure, polarization 1 and 2 is referred to as 0° and 90°, respectively. It is evident from the plot that the super cell is capable of capturing the microwave energy and converting around ${\rm{70 \% }}$ of the collected energy to usable DC power across the load resistance. Moreover, the profile of the efficiency curves for all incident angles overlap having a peak efficiency at a frequency of 2.1 GHz. This is very critical as the main goal of a multi-polarized harvester is to capture the incident energy maximally from different angles at a specific or a range of frequencies. The measured optimal frequency has a slight deviation from the designed one due to fabrication errors. Since the behaviour of the diode is a strong function of the resonance frequency, the input power and the load resistance, a slight alteration in the resonance frequency can also cause a shift in the optimal input power level and the optimal load resistance. In the measurement, the peak received power occurred at a resonance frequency slightly different from the one predicted in the simulation. This frequency shift changed the optimal operating point of the diode in terms of power level and load resistance. As a results the efficiency at different input power values has also changed as observed from the curves obtained in the measurement (Fig. 8) compared to that obtained in the simulation results (Fig. 10(a)).

**Figure 12:**
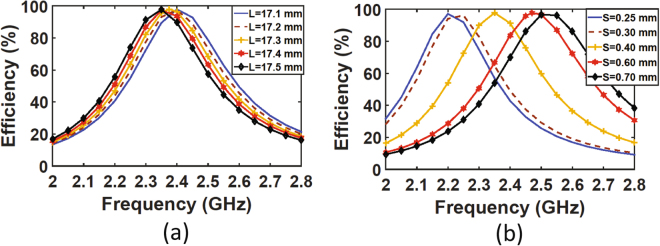
Simulation results of the radiation to AC efficiency as a function of (**a**) the length, L, of the sub-unit cell and, (**b**) the separation distance, S, between two adjacent sub-unit cells.

The resonance frequency is strongly affected by the length of the sub-cell, L, and most importantly by the separation distance between the cells, S. To understand how a small deviation in the fabrication of the metasurface can cause a frequency shift as experienced in the measurement, a sweep of different values of L and S as a function of the total efficiency of the super cell was performed numerically. Figure 12(a) shows that when the length of the cell is slightly changed from the designed length of 17 mm to values ranging between 17.1 mm to 17.5 mm, the resonance frequency is shifted to lower values. This deviation increases as the length L increases. However, a more pronounced shift is observed when a slight deviation in the separation distance between the sub-unit cells occurs as observed in Fig. 12(b).

## Discussion

**Figure 13:**
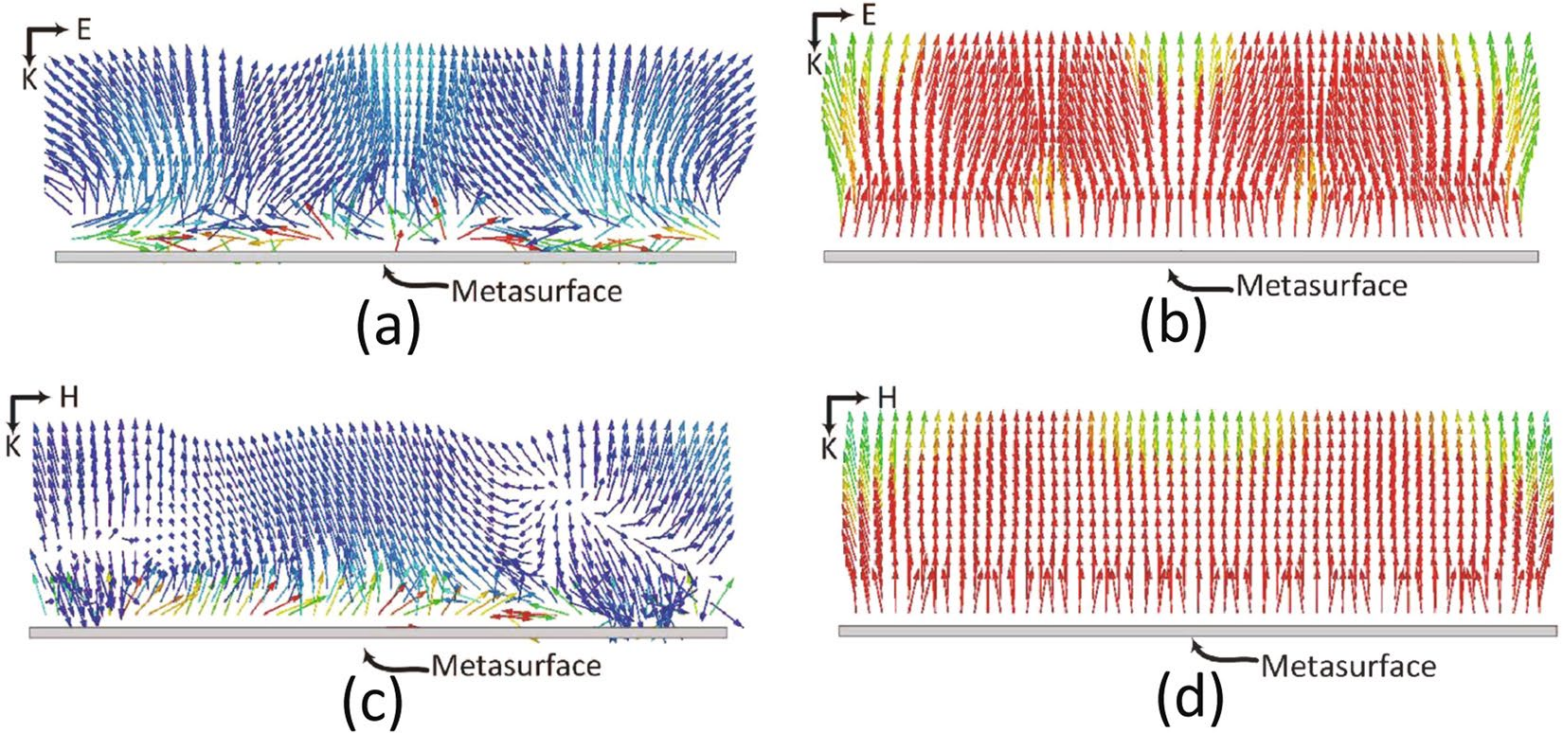
The simulation results of the $12\times 12$ cells array showing the Poynting vector distribution over a rectangular cross section perpendicular to the plane of the array for (**a**) polarization 1 at the resonance frequency of 2.4 GHz (**b**) polarization 1 at a frequency of 4 GHz (away from resonance). (**c**) polarization 2 at the resonance frequency of 2.4 GHz, and (**d**) polarization 2 at a frequency of 4 GHz. Darkest blue corresponds to 0 mW/m^2^ and darkest red corresponds to 1 mW/m^2^.

When an infinite array of the super cells shown in Fig. 1 is arranged in a periodic fashion, a surface is created with an impedance matched to the free-space impedance. Such feature is the key advantage to the current design and the main reason that enable metasurfaces to be an excellent candidate for electromagnetic energy harvesting. When an incident planewave strikes a metasurface that is matched to the free-space impedance, almost all the energy is absorbed within the metasurface harvester with minimal energy scattered away from the interface of the structure. To illustrate this concept, a $12\times 12$ cells with nine super cells were simulated numerically. In the simulation, the metasurface was excited by a planewave with two polarizations as depicted in Fig. 1. The metasurface was enclosed by an absorbing boundary condition to ensure no power is reflected back to the metasurface from the boundary walls. Figure 13 shows a rectangular cross section of the Poynting vector perpendicular to the metasurface array for both polarizations. The range of colors represents the magnitude of the energy flux ranging between the lowest of 0 mW/m^2^ (deep blue) to the largest of 1 mW/m^2^ (deep red). When the plane wave hits the metasurface at the resonance frequency of 2.4 GHz (see Fig. 13(a) for polarization 1 and Fig. 13(c) for polarization 2), the energy is absorbed by the cells as depicted by the red color close to the metasurface interface. In addition, minimal energy is scattered/reflected away from the surface as illustrated by the blue colored arrows pointing away from the surface. This indicates that the energy is absorbed within the metasurface array at the resonance frequency. Away from the resonance frequency (i.e., 4 GHz), however, the metasurface acts as a reflector as depicted by the red colored arrows pointing away from the metasurface as shown in Fig. 13(b) for polarization 1 and Fig. 13(d) for polarization 2.

Although the designed metasurface at the resonance frequency experiences full absorption, this does not necessarily qualify it as an energy harvester. This is due to the fact that the absorbed energy can be lost within the dielectric substrate as the case in previous metasurface absorbers presented in the literature^13^. In metamaterial absorbers, the main goal is to absorb the energy regardless of where the absorbed energy is lost. However, in metamaterial harvesters, the metasurface must be able to: (1) absorb all the energy from the incoming wave, (2) deliver all the absorbed energy to the load, and (3) replace the load with a rectification circuitry and convert the collected AC energy to DC. From Fig. 2(a) and (b) it is evident that point (1) and (2) are satisfied. Minimizing the rectification losses (point (3)) remains a challenge due to the inevitable losses of the diode during the turn on process. In addition, the generation of harmonics from the nonlinearity of the diode is another source of losses. Although it is beyond the scope of the present study, it is suspected that such losses can be minimized by introducing filters to suppress the energy lost due to higher order harmonics.

In previous works, rectennas were presented as a single element or an array. Various antenna types were used to receive the microwave energy and convert it to AC power such as dipoles^22^, patches^23^, bowties^24^, loops^25^ and spirals^26^. The AC power is then fed to a rectifier which can be half wave series^27^ or parallel diode^28^, or full wave rectifier^29^. The total efficiency of a single rectenna is typically higher than an array of rectennas. This is mainly due to the energy lost by the AC or DC channeling circuit, which combines the power collected by all the elements in the rectenna system and channels it to a single load. In addition, the effective aperture of a single antenna is typically much larger than its physical footprint. A number of rectenna arrays were presented in the literature for linear and circular polarization. In^30^ a rectenna array made of three elements semicircular slotted antennas operating at 9.3 GHz was presented. The array achieved 21% radiation to DC efficiency when illuminated by a field with a power level density of 245 $\mu W/c{m}^{2}$. A differential rectenna array with patch antenna elements connected in series and parallel connection operation at 5.8 GHz was proposed in^31^. A maximum efficiency of 44% was obtained with an input power level of −11 dBm. A dual band rectenna consisting of 1 × 4 quasi-Yagi antenna array was used in^32^ to harvest the microwave energy at 1.85 GHz and 2.15 GHz. The array captured the incoming electromagnetic energy with an efficiency of 40%. In^33^, a four element circularly polarized patch antenna array was used as a rectenna system operating at 24 GHz. The total radiation to DC efficiency was 24% at a power level of 10 $mW/c{m}^{2}$. In^34^, a large scale rectenna system consisting of 2304 rectennas was tested experimentally. The array was able to convert and channel 50% of 1.2 kW input power to a load resistance of 358 ${\rm{\Omega }}$. A linearly polarized 7 element patch antenna array connected to a rectifier with three-stage Dickson charge pump circuit was presented in^35^. A measured efficiency of 41% was realized with an input power of 10 dBm. A broadband rectenna array made of spiral antennas with diodes mounted at the feed of each antenna was studied in^36^. The energy was collected over the frequency range of 6–15 GHz with efficiencies varying between 5 and 45%. A rectenna array made of dipole antennas integrated with fullwave rectification was presented in^29^ with a reported radiation to DC efficiency of 52%. For brevity and clarity, a number of papers presented in the literature in terms of efficiency, polarization, bandwidth, power level and operating frequency are summarized in Table 2. In all previously presented rectenna arrays, the target was either linear or circular polarization. However, this paper focuses on harvesting the energy with multi-polarizations, achieving a high radiation to DC efficiency of 70%.

**Table 2:** A summary of rectenna arrays presented in the literature.

Paper reference	Feature				
	Polarization	Bandwidth	Power level	Operating frequency (GHz)	Efficiency (%)
30	Linear	Narrow	245 $\mu W/c{m}^{2}$	9.3	21
31	Linear	Narrow	0.041 $W/c{m}^{2}$	5.8	44
32	Linear	Narrow	455 $\mu W/{m}^{2}$	1.85 and 2.15	40
33	Circular	Narrow	10 $mW/c{m}^{2}$	24	42
34	Linear	Narrow	1.2 kW	2.45	50
35	Linear	Narrow	10 dBm	0.915	41
36	Circular	Wideband	1–1.6 $mW/c{m}^{2}$	6–15	5–45
29	Linear	Narrow	0.15 $mW/c{m}^{2}$	2.4	52
19	Circular	Narrow	13 $mW/c{m}^{2}$	5.8	74
21	Linear	Narrow	12 dBm	2.82	40
37	Linear	Narrow	30 mW	3.4	76
This paper	**Multi-polarized**	Narrow	9 dBm	2.1	70

## Methods

### Simulation

**Figure 14:**
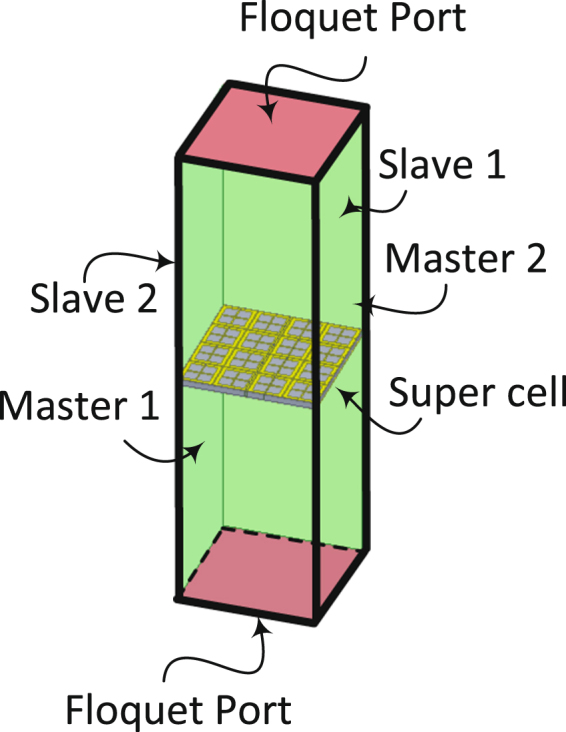
An illustration to clarify the periodic boundary condition simulation setup of the super cell in HFSS showing the 4 master/slave boundary walls and the 2 Floquet ports.

The radiation-to-AC efficiency simulation of the super cell was performed using the full-wave numerical simulation tool HFSS. In the simulation the super cell was placed inside a waveguide with periodic boundary condition assignment on the lateral walls. Master and slave periodic boundary conditions were used for the four lateral walls to simulate the super cell in the presence of an infinite array as shown in Fig. 14. The top and bottom sides of the waveguide were assigned with Floquet excitation ports with modes that support the two proposed polarizations illustrated in Fig. 1. The load resistors were modeled as a lossy sheet and the energy dissipated across each resistor was calculated using the surface loss density formula embedded in the simulator’s calculator. The simulation were performed in two stages: first, with reference to Fig. 1, each cell was terminated by a resistor having a resistance value of 175 Ω. Then, the super cell was studied in terms of power absorption and power dissipation across the total 16 resistors. Then, a feeding network was placed at a different layer and the energy from each cell was channelled to the feeding network through via holes running through the two substrate and the ground plane. The second stage of the simulation was performed on the super cell including the feeding network. The energy captured by each 8 cells (having the same polarization) was channelled to one feeding network. Hence each super cell contains two channeling networks, one network for each polarization as depicted in Fig. 4. At the end of each feeding network a resistive sheet is placed between the end of the feeding line and the ground plane. The super cell along with the feeding network was simulated in the transmitting mode by replacing the Floquet excitation ports in Fig. 14 with an absorbing boundary conduction. In addition, the two resistive sheets at the end of each feed line were assigned excitation lumped ports with input impedance of 175 Ω.

**Figure 15:**
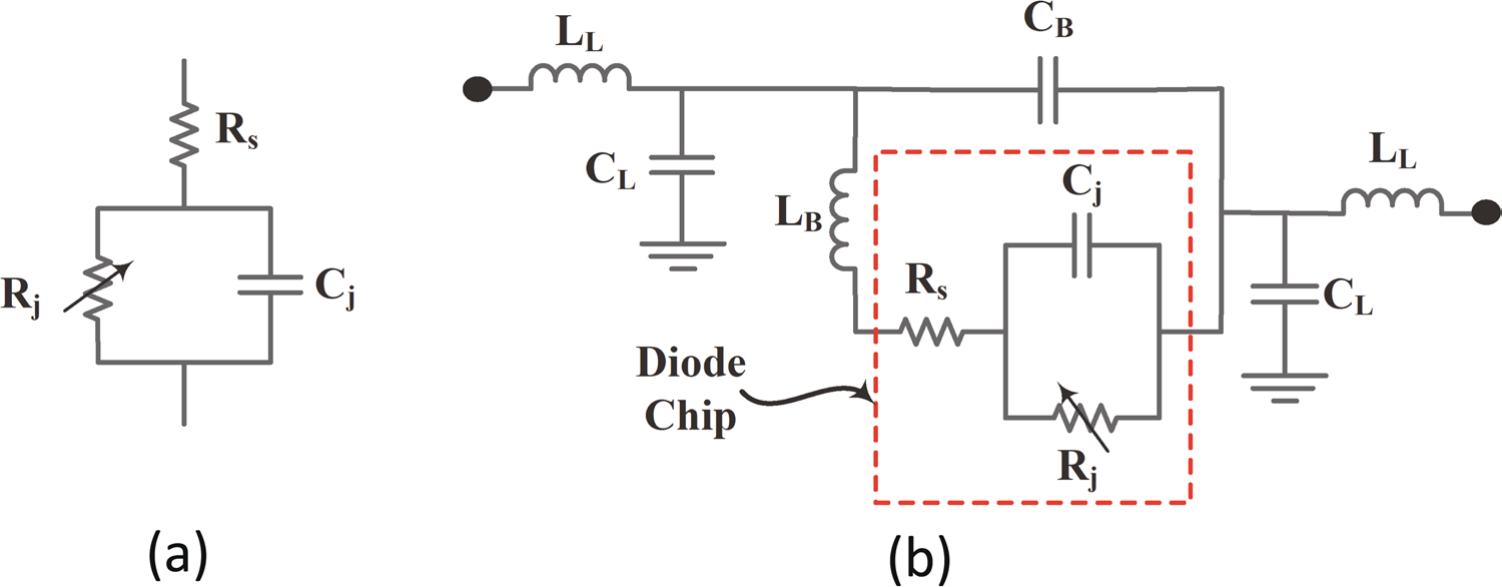
Circuit model for (**a**) the diode chip, and (**b**) the diode chip including the parasitic inductances and capacitances of the packaging, leadframe and the bondwires^38^.

The AC to DC simulation of the rectifier was performed using the circuit simulator ADS. First, the impedance matrix of the super cell obtained for each polarization in the transmitting mode analysis from the full-wave simulation was extracted and saved in a citi file format. Here the Z matrix is refereed to as the directional impedance at the feed location circled in Fig. 4 for each polarization. Then the file is imported in ADS and saved in a DAC component. A single tone frequency power source was used with an internal frequency dependant impedance identical to the super cell for each polarization. This will ensure that the rectifier is energized by a source that have the same impedance as the super cell’s input impedance and hence impedance matching between the super cell and the rectifier can be performed. The HSMS 2860 Schottky diode chip can be modeled with its series resistance ${R}_{S}$ in series with a parallel combination of the diode’s junction capacitance ${C}_{j}$ and junction resistance ${R}_{j}$ as illustrated in Fig. 15(a). However, to account for the parasitic capacitances and inductances due to the bondwire, leadframe and the diode packaging, the diode model can be extended to the circuit shown in Fig. 15(b). In the circuit model, ${L}_{L}$ is the leadframe inductance, ${C}_{L}$ is the leadframe capacitance and ${L}_{B}$ is the inductance due to the bondwires^38^. The rectifier was analysed using the LSSP and HB simulators to account for the nonlinearity effect of the diode. Then two different rectifiers were designed accordingly and the traces for the rectifier were added after the feed location for each polarization (see Fig. 4) before the fabrication stage.

### Fabrication

**Figure 16:**
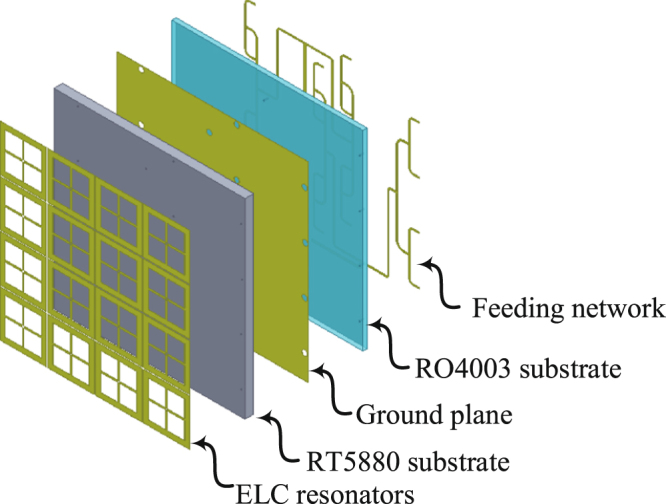
A schematic showing an exploded view of the 3 layers and the two substrates within a super cell. In addition, 16 via holes were used to connect the resonator layer with the feeding network layer.

In the fabrication stage, a $3\times 3$ super cell array was fabrication which contains $12\times 12$ cells. The full system consists of 3 different layers and two substrates as shown in Fig. 16 for an exploded view of a single super cell. The first layer consists of the resonators which are hosted on top of the RT5880 Rogers dielectric material. Then a ground plane is sandwiched between the two dielectric materials (RT5880) and (RO4003). The feeding network along with the rectifiers were placed at the third layer hosted at the bottom of the RO4003 dielectric substrate. The ground plane contains circular holes to ensure that the vias running through the holes (connecting the resonators at layer 1 to the feeding network at layer 3) do not touch the ground plane. The final fabricated system is shown in Fig. 9(a) and (b).

## Conclusion

We demonstrated the design of a microwave energy harvester that is capable of scavenging energy from multiple polarizations. A super cell consisting of $4\times 4$ cells was designed and analysed for maximum radiation-to-AC conversion efficiency. The collected energy was guided to the load resistance through a hybrid channeling mechanism that combines AC and DC channeling networks. A rectifier was then designed and attached along with a feeding network at a different layer to convert the collected AC power to DC. The obtained measurement results agree well with the simulated ones aside from a slight shift in the resonance frequency due to fabrication error. Although the proposed energy harvester showed excellent ability to capture the incoming energy from various polarizations, it suffers from a narrow bandwidth. Future papers will address this issue.
